# AI‐Driven Hemodynamic Detection of Self‐Induced Daydreaming With EMG‐Based Physiological Triggers During Pre‐ and Post‐Prandial States Using fNIRS and EGG

**DOI:** 10.1002/cns.70899

**Published:** 2026-05-01

**Authors:** Anusha Ishtiaq, Zia Mohy‐Ud‐Din, Abdullah Al Aishan, Noman Naseer, Syed Ghufran Khalid, Jahan Zeb Gul

**Affiliations:** ^1^ Department of Biomedical Engineering Air University Islamabad Pakistan; ^2^ Department of Basic Medical Sciences College of Applied Medical Sciences, King Khalid University Abha Saudi Arabia; ^3^ Department of Mechatronics Engineering Air University Islamabad Pakistan; ^4^ Department of Engineering Nottingham Trent University Nottingham UK; ^5^ Department of Electronic Engineering Maynooth University Maynooth Ireland

**Keywords:** AI‐based detection, daydreaming, EGG, EMG trigger, fNIRS, ML classifiers, prandial state

## Abstract

**Background:**

Daydreaming can be monitored either to avoid it while doing hands‐on tasks or to enhance it to foster creativity. Although significant research has been conducted in Brain recordings and Machine learning, some problems have not received sufficient attention. One of them is the automated identification and classification of daydream states with emphasis on physiological signals and prandial states. Until now, researchers have been relying only on subjective questionnaire‐based methods of daydream identification, neglecting neural hemodynamics.

**Methodology:**

In this study, EMG‐based physiological triggers have been incorporated to detect self‐induced daydream episodes in pre‐ and post‐meal prandial states. For the AI‐driven hemodynamic monitoring of the brain in relation to the analysis of the electrical activity of the stomach during self‐induced daydreaming, fNIRS and EGG signals of 30 participants were recorded, preprocessed, and investigated simultaneously. Both the duration and frequency of the daydreaming episodes were analyzed using these two modalities, which were further subjected to a feature extraction and class label encoding process to facilitate a four‐class classification of daydreaming and prandial state.

**Results and Conclusion:**

Machine learning models were incorporated for classification and resulted in the highest testing accuracy of 90.77% for daydream detection and gave insights into the connection between meal consumption and daydreaming.

**Future Work:**

In the future, this study could serve as the preliminary basis for multimodal monitoring systems used to assess the state of cognition in parallel with the analysis of meal intake patterns. This research can also lead to the development of person‐specific treatments in the domain of mental and attentional health.

## Graphical abstract

(A) Problem analysis and its consequences: In a non‐DD State, chewing motor activity increases and brain cognitive activity decreases and in a DD State, chewing motor activity reduces and brain cognitive activity increases. Moreover, in the pre‐prandial state, DD episodes and frequency increases, and in the post‐prandial state, DD episodes and frequency decreases. (B) Data acquisition protocol involves EGG, EMG, and fNIRS signal acquisition electrodes placement on the stomach region, temporal region, and head respectively. (C) Hybrid data processing and analysis of EMG, fNIRS, and EGG signals during the paradigm. (D) Machine algorithms for EGG‐based prandial state classification and further feature selection of the fNIRS signals for daydream classification. Moreover, the label set transformation technique was applied to study the effect of meal intake on daydream classification.

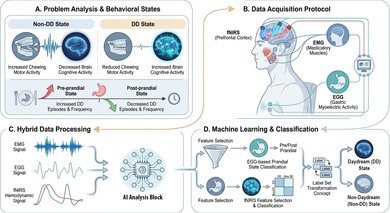

## 1 Introduction

Our attention naturally shifts between the real environment and internal thoughts. These intermediate episodes of imagination are termed daydreaming (DD). This neural activity is described and differentiated from mind wandering (MW) by Emily et al. [1], Hagar et al. [2], and Halleyson et al. [3]. DD has pros and cons. According to the study references, mind wandering is the loss of attention and thinking about random things. On the other hand, DD is a focused thinking activity in which the person purposefully thinks about something. On the positive side, DD promotes creativity, improves problem‐solving capabilities, aids memory consolidation, and promotes mental relaxation by taking the mind elsewhere from daily worries [4]. It can also help people to think about the future and about what they want to achieve. Yet, too much DD, called maladaptive daydreaming (MD), can result in distraction, decreased productivity, problems in concentrating on important work, and sometimes may even lead to avoidant behavior, which entails neglecting reality. Thus, unchecked or excessive DD may come into conflict with daily life; hence, monitoring and the investigation of its link with daydream factors is necessary to be studied.

Epidemiological studies indicate that approximately 2.4%–2.5% of individuals in the general population fulfill the criteria for clinically significant maladaptive daydreaming, whereas among younger adults (for instance, those aged 18–30), prevalence rates can increase to 5.5%–8.5% in certain samples [5, 6]. In particular populations, the incidence can be significantly greater. For instance, studies conducted with medical students in Sudan and Saudi Arabia revealed that 34% and 70%, respectively, recognized themselves as maladaptive daydreamers [7]. Consequently, MD reduces real‐world engagement and can disrupt academic, social, and work‐related functioning [5]. Individuals with MD commonly exhibit elevated rates of accompanying psychiatric symptoms, including ADHD, depression, anxiety, and obsessive‐compulsive characteristics. Intricate profiles of comorbidity are prevalent; a clinical study revealed that more than 74% met the criteria for three or more additional psychiatric conditions [8].

MD impacts daily activities; studies show that MD lowers quality of life and is linked to feelings of loneliness, psychological distress, negative emotions, diminished self‐esteem, and weakened emotional regulation [9]. Students experiencing more severe MD symptoms tend to have reduced academic performance (e.g., GPA), indicating functional difficulties in their education [10]. Because of the high comorbidity of MD with other mental health disorders, it raises the overall complexity of psychiatric conditions, typically linked to increased cost with utilization of mental health services, medications, and therapies [8].

For daydream detection, some conventional methods are being used, such as self‐report, survey, and questionnaire methodologies, which are common in DD studies because they provide direct access to an individual's subjective experience. The measurements of such tools are often based on standardized scales, for example, the Mind‐Wandering Questionnaire (MWQ) in the study of Igor et al. [11] and Metin et al. [12], and on the basis of the study by Sameera et al. [13], the Daydream Frequency Scale (DFS), and the Imaginal Processes Inventory (IPI), which assess how DD occurs, what it consists of, and how it is regulated. These are all subjective methods, and, on the one hand, they provide easy‐to‐use tools for exploring patterns of spontaneous thought and MW, but, on the other hand, they do not go beyond the information available from the subject's feedback. Like, instead of just relying on subjective techniques, we can explore the applicability of physiological trigger‐based daydream detection.

For such physiological triggers, some studies suggest that motor activity is suppressed when a cognitive task is being performed [14, 15]. These findings are supported by the experiments of Zmeykina et al. [16] and Escan et al. [17]. This validates the inhibition of the motor cortex when alpha and theta frequency waves of the EEG are elevated during the activation of the brain's cognitive region. Hence, the motor task‐based trigger would be useful to detect the activation of the cognitive region, especially during DD. Although no such trigger has been used according to present studies, there do exist examples of EMG‐based trigger mechanisms for EEG signals analysis, such as Gao et al. [18] and Artoni et al. [19] have investigated the coupling between EMG trigger and EEG signals. Thus, EMG can serve as a trigger for monitoring neural signals during DD.

Brain states such as DD or focus, as well as other states of mental fatigue or cognitive engagement, can be investigated with various neurophysiologic recording techniques [20, 21, 22, 23]. To investigate neural activity during DD, biomedical imaging techniques like fMRI, EEG, etc., have been utilized till now. In one study, Chutimon et al. [24] have used an EEG scan to identify decoupling of attention on the basis of patterns of neural responses related to inattention. The results showed that the SVM‐based learning model, in combination with higher‐level feature extraction, provides the best classification accuracy of 75%, which confirms the reliability of the EEG signal as a measure of internal cognitive states, once processed correctly.

In another study of Xiaodong et al. [25], DD was referred to as a type of cognitive “noise” that decreases the accuracy with which mental activities can be classified according to the EEG signal. To alleviate this problem, they detected and separated a type of DD states solely from the EEG when treating DD as an independent cognitive state. Similarly, several research papers, including those of Henry et al. [26], have tried to discriminate Mind‐wandering (MW) by using EEG, and they have employed SVM and Logistic Regression for that purpose. Likewise, the research of Shaohua et al. [27] attempted to identify MW in subjects, let them watch video lectures along with an 8‐channel EEG acquisition to identify segments of inattention, performing a novel experiment with guided learning as well as future planning tasks. Similarly, a recent fMRI study has revealed that DD and creativity share a common neurobiological basis, which occurs as a process of the default mode network (DMN) [28]. This network is also recruited during spontaneous thought, that is, imagination and memory retrieval, as well as mind‐wandering. The DMN lights up in the same way on fMRI imaging when people are engaged in creative thought or DD intensely.

Additionally, according to the findings of Jungru et al. [29] fNIRS was used for the detection of brain function using levels of Oxygenated hemoglobin (HbO). The investigators based their study on the idea that neural activity induces an increase in regional cerebral blood flow (rCBF), leading to a rise in the concentration of HbO in activated cortical areas. Recently, Yuyun et al. [30] have studied mental fatigue through analyzing fNIRS signals. Besides, in a recent work of Jin et al. [31], they also employed fNIRS for machine learning models for classifying cognitive states. In one study, fNIRS has been utilized to monitor mind‐wandering [32] and classify it using a machine learning technique [33]. The highest F1‐score of 73% has been achieved in that study. Thus, fNIRS has been utilized in a few experiments for the monitoring of MW but not the induced DD.

Along with the detection of DD, the factors affecting this neural activity also need to be analyzed for further applications. Many factors control the patterns of DD episodes, such as cognitive variables, creativity, psychological feelings, etc. One of the most important physiological factors involved in daydream control is the prandial status that contributes to the shift into internal thinking [34]. For this investigation, Jan et al. [35] conducted a study. Their results suggest that during the hunger state, more frequent episodes of MW are observed. Thus, along with DD, to examine the prandial state, a technique needs to be utilized that captures gastric movements well. Electrogastrography (EGG) is a non‐invasive method for gauging gastric myoelectric activity [36]. A system EGG DWPack system [37] has also been utilized to collect recordings of EGG signals in order to study the gastric activity, both in the fasting state (premeal) and in the prandial (post‐meal) paradigm. The primary focus of this analysis was on the comparison between the physiologic recordings of pre‐ and post‐prandial states, as well as the characterization of various gastric rhythms and amplitude‐based features [37, 38].

Different EGG recording techniques and signal processing methods are available [39]. In reference to our interest in stomach states classification, the study performed by M. Faizal et al. [40] compared five features for separating pre‐prandial from postprandial states. They tested two classifiers, SVM and ANN, and the highest classification accuracy of 82.3% was given by the SVM classifier.

Still, none of the research is available on monitoring the effect of the prandial state while performing daydream tasks. Furthermore, no study to date has investigated the changes in HbO levels of the brain during DD, keeping the prandial state under consideration. Now, there is a need to systematically investigate how nutritional state can have an impact on self‐induced daydreaming (SiDD). Moreover, apart from relying on self‐reported questionnaires only, DD episodes need to be detected via some physiological trigger‐based logical mechanism.

This paper proposes an AI‐driven Hemodynamic detection framework to monitor self‐induced DD with the help of EMG‐based physiological triggers during pre‐ and post‐prandial states. For this purpose, fNIRS and EGG signals have been acquired, processed, and used to classify DD states and prandial status, respectively. The changes in HbO levels of the brain have been investigated while DD, keeping the prandial state under consideration, to classify DD using artificial intelligence (AI) models. Moreover, a hybrid dataset has been designed using Label Powerset Transformation (LPT) and optimized through channel and feature selection of the fNIRS data.

## 2 Methodology

**FIGURE 1:**
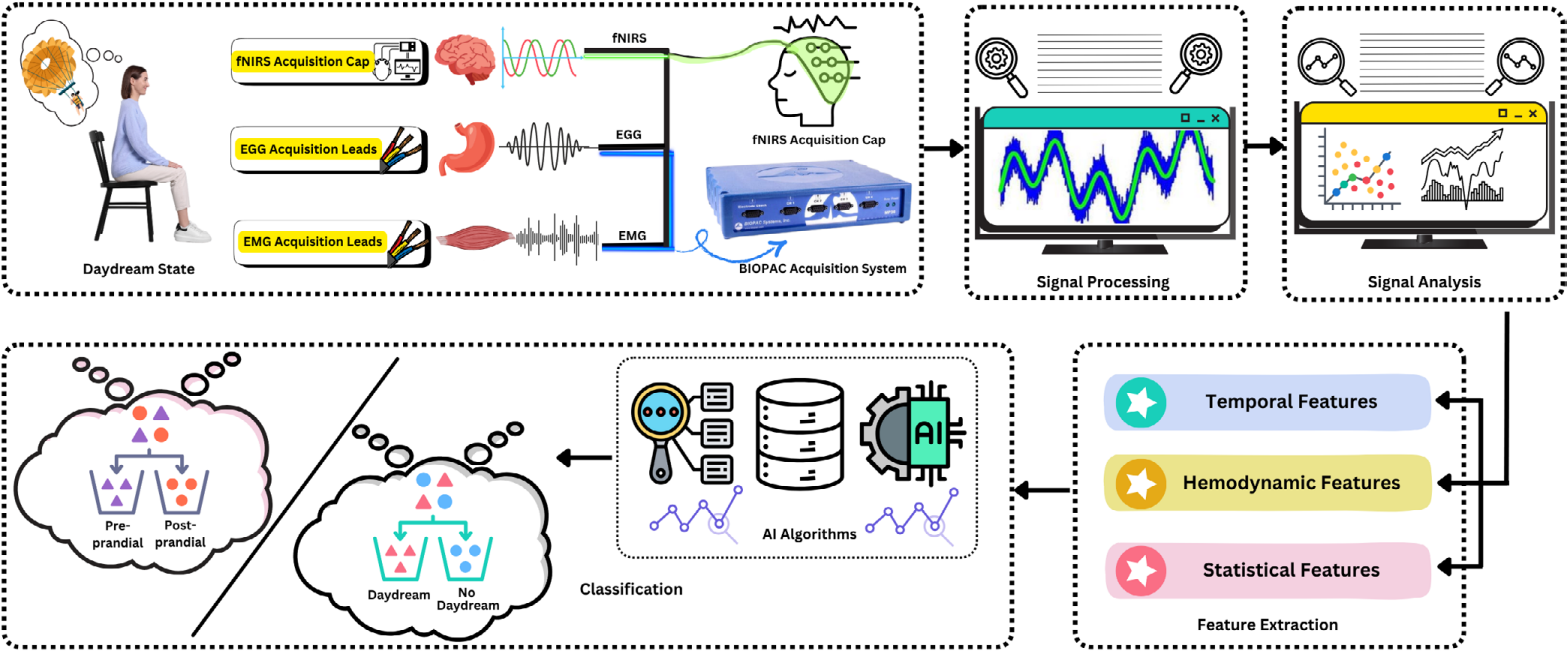
Basic block diagram showcasing the flow of this study from data acquisition to classification of DD v/s non‐DD class and Pre‐prandial vs. Post‐prandial Scenario. The acquired signals are processed and analyzed, and their features are fed into the ML Classifiers.

This work presents the development of AI‐based Hemodynamic detection of self‐induced DD using EMG physiological triggers during pre‐ and post‐prandial states through fNIRS and EGG signals. For this purpose, the study follows the pipeline of data acquisition, signal processing, data analysis, feature extraction, classification, and results evaluation. Similar basic steps have been followed for both the daydream identification and the prandial state detection, as depicted in the Figure 1.

### 2.1 EMG‐Based Physiological Trigger

**FIGURE 2:**
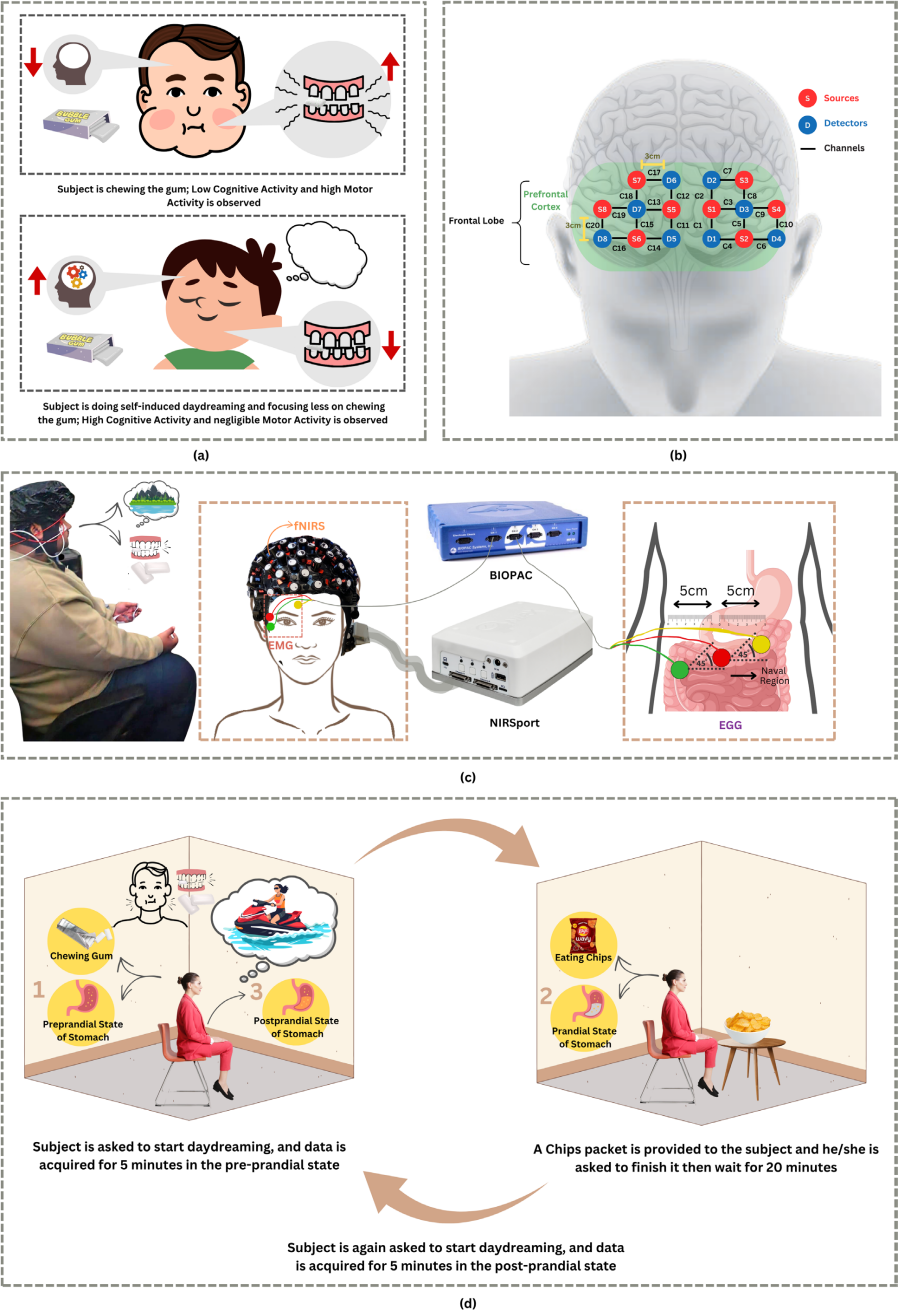
Data acquisition protocol: (a) EMG‐based physiological trigger to detect bruxism; (b) fNIRS Cap Montage design for channels; (c) electrode Placement for EMG, fNIRS, and EGG signals; (d) experimental paradigm includes pre‐prandial and post‐prandial data acquisition, where a meal is served in between.

During DD, with the shift of attention toward internal thoughts, motor activity is reduced to a negligible level. This is in accordance with the evidence that the motor cortex is suppressed when the cognitive region is activated [16, 17]. To detect such a suppression of motor activity, the facial muscles serve as a good monitoring site for EMG recording during motor task‐based paradigms like chewing. Hence, in this study, a reliable stimulus‐based DD detection mechanism was designed, for which the chewing activity was targeted, as illustrated in Figure 2a. This is because it has been used for the effective detection of facial movements, specifically the chewing task [41].

### 2.2 Paradigm Design

The physiological signals, EMG, fNIRS, and EGG, of 30 participants (15 males and 15 females) have been acquired. All subjects were university students belonging to different regions of the country. Ethical clearance from the institutional review board was obtained for this study, and it adhered to the guidelines of the Declaration of Helsinki. A consent form was signed by the subjects before the study. The participants were chosen according to the inclusion criterion shown in Table 1. They were instructed about the full procedure, and they were then asked whether they were fasting for at least 5 h. Chewing gum was provided to the participants to track the chewing activity for the trigger‐based DD detection, as depicted in Figure 2a. In the paradigm used, and shown in Figure 2d, 5 min of data were acquired in the fasting state; food was then served, and there was a 20‐min break, followed by another 5 min of data acquisition.

**TABLE 1:** Inclusion/Exclusion criteria of the participants for this study of daydream and prandial state detection.

Condition	Inclusion criterion
Fasting	Minimum 5 h
Caffeine intake	No (at least 4 h earlier)
Hair	Non‐greasy and non‐colored
Body weight	Normal (as per BMI of 18–25 range)
Blood pressure	120 mm of Hg systolic by 80 mm of Hg diastolic
Mental health	No psychological or mental issues
Age group	18–30 years

Every single participant consumed chips of 500 kCal. Using the same common food for each subject in a study having the same caloric value allows for uniformity and reduces variability of the diet, which is an issue when it is necessary to control for potential outcomes. Such a method makes it possible to clarify the influence of meal intake on physiological or cognitive signals—for example, EGG/fNIRS waveforms.

### 2.3 Physiological Data Acquisition

Physiological signals of fNIRS, EMG, and EGG have been acquired from 30 participants on the basis of a defined paradigm.

#### 2.3.1 Data Acquisition of EMG Signals

To detect the chewing activity, EMG of the temporalis muscle was recorded because this muscle responds well to the chewing action [41]. Another alternative could have been the masseter muscle, but in most male subjects, the presence of a beard resulted in noisy data of masseter EMG. So, the temporalis muscle was given the preference in this study, as depicted in Figure 2c, and electrodes were placed over the right temporalis muscle and grounded onto the forehead. Therefore, single‐channel EMG was obtained for all individuals.

When a person is indulged in intense internal thoughts, he disconnects with the external environment where he was chewing; hence, the chewing activity slows down or stops entirely. There was a very noticeable reduction in chewing since the motor coordination necessary for rhythmic mastication was reduced when neural resources were drawn out and redirected again to the internal mental state, as illustrated in Figure 2a. In numerous cases, this reduction progressed to the point of cessation of the chewing movement, and it was correlated with the beginning of the SiDD stage. Hence, the trigger of chewing activity was essential to track DD episodes.

The subjects chewed mainly on the right side, so the right temporalis muscle was suitable for observing the side priorities of their chewing, as indicated in Figure 2c. The recording was performed using a BIOPAC MP36 acquisition system with a sampling frequency (Fs) of 2000 Hz, band pass frequency range of 30–500 Hz [42, 43] and a Notch filter of 50 Hz to remove power line noise.

#### 2.3.2 Data Acquisition of EGG Signals

For electrogastrogram (EGG) recording, single‐channel electrodes were placed over the abdomen (stomach region) for the recording of slow wave electrical activity of the stomach (gastric rhythms). The arrangement of electrodes corresponded diagonally to the stomach area, and the center was set on the navel, which was the reference point. The positive electrode was set to the left side of the abdominal wall, and the negative electrode was placed diagonally on the right convex below the navel, as shown in Figure 2c. EGG signals were recorded using the BIOPAC MP36 acquisition system (2000 Hz of sampling frequency) with band pass frequency limits of 0.001 and 10 Hz and a Notch filter of 50 Hz to remove power line noise. As EGG contains very low frequency components, the frequency range is limited to a set less than 10 Hz in the low‐pass filter (LPF) because of limitations of the BIOPAC acquisition system.

#### 2.3.3 Data Acquisition of FNIRS Signals

fNIRS is a non‐invasive optical neuroimaging technique that quantifies brain activity by variations in the concentrations of HbO and deoxy‐hemoglobin (HbR) in the brain cortex [23, 44, 45]. It operates by shining near‐infrared light through the scalp and skull, where it is absorbed or reflected by the tissue beneath. Because neural activation causes a change in blood oxygen level in the corresponding area of the brain (neurovascular coupling), the fNIRS data are a valid but indirect monitoring of brain activity.

Hemodynamic brain signals were recorded with an fNIRS cap connected to the NIRSport system with 20 channels located on the prefrontal cortex for a neurophysiological measure of DD presented in Figure 2b,c.

The prefrontal cortex was the focus because of its involvement in executive processes, self‐referential thinking, and the default mode network that is involved during internally directed states such as MW and DD. The calculated cap setting [46, 47, 48, 49, 50] was carefully proposed on the basis of normal anatomy landmarks to place it on all subjects with firm, accurate positioning, as well as to reliably measure changes of hemodynamics (change of HbO) from the right and left frontal sites throughout the experiment period. NIRS port Sampling Frequency (Fs) was set to 10.17 Hz, and the band pass filter was 0.015–0.4 Hz.

### 2.4 Signal Processing

Before signal analysis and classification, fNIRS and EGG signals needed to be processed to extract meaningful information. EMG signals were only used as the daydream detecting trigger; hence, their processing was not required.

#### 2.4.1 Data Conditioning of fNIRS Signals

**FIGURE 3:**
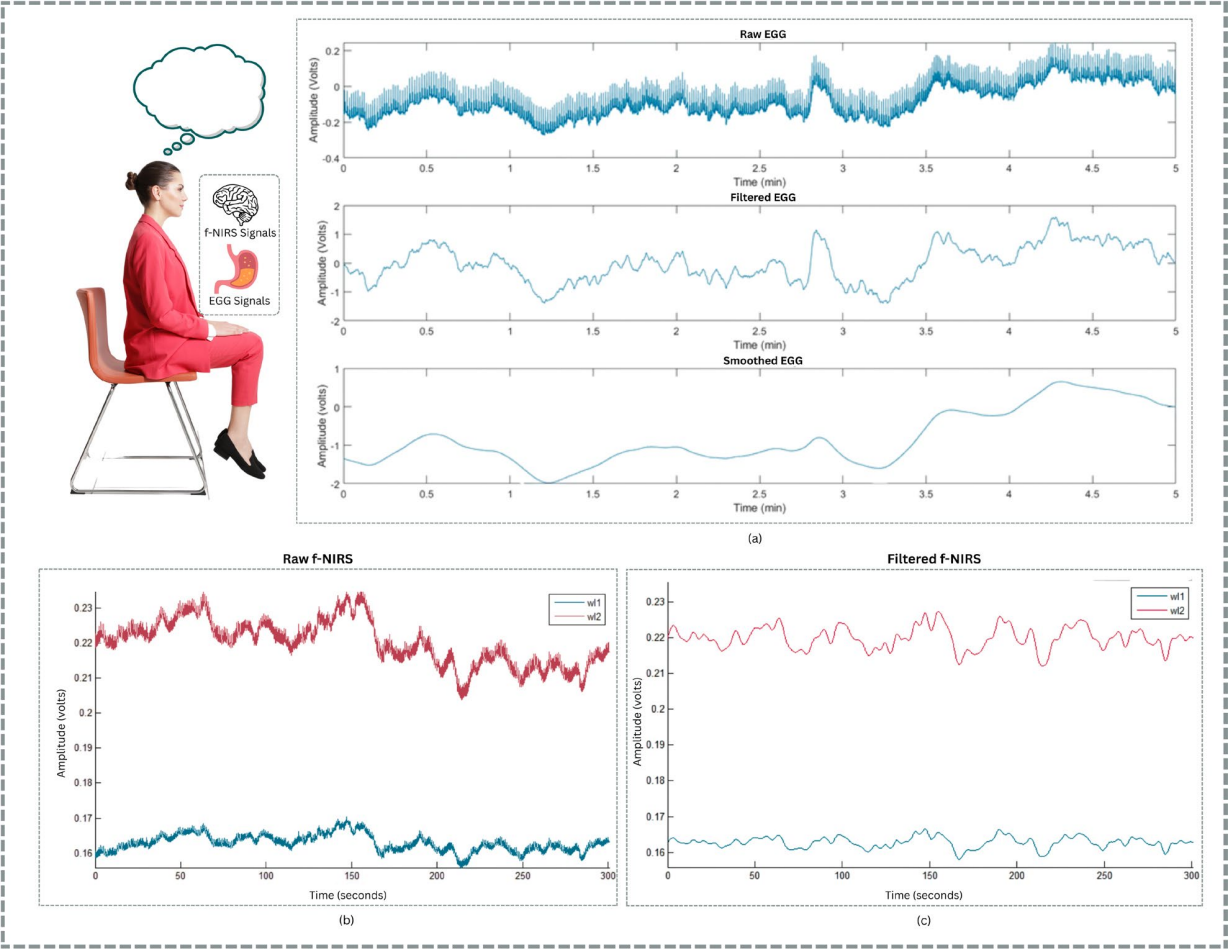
Signal processing: (a) Filtering and Smoothing of EGG signals; (b) raw fNIRS signals of wavelength 1 (wl1) of 760 nm and wavelength 2 (wl2) of 850 nm; (c) filtered fNIRS signals of both wavelengths.

The pre‐processing for the fNIRS signal was performed using the NIRSlab software. Raw. nirs files were imported and band‐pass filtered in the range 0.01–0.4 Hz to focus on the hemodynamic signals, removing very slow trends and high‐frequency noises (e.g., cardiac and respiration artifacts), as shown in Figure 3b,c. Post‐preprocessing, the hemodynamic responses, that is, HbO concentrations, were employed to measure brain activation. Channel connection was also checked within NIRSlab to make sure the signal was noise‐free, and all channels were connected and working properly. This would have helped to identify any noisy, flat, or disconnected channels that may have resulted from poorly placed or connected optodes. However, there were no bad connections, and therefore all channels were retained in the final files. Finally, the filtered data was stored as. csv files, which were easily imported for further analysis and feature extraction in other environments (e.g., using Matlab and Python).

#### 2.4.2 Data Conditioning of EGG Signals

In the recent review article by David et al. [39], methods for EGG acquisition show slight variations in electrode placement, sampling rate (fs), filtering methods, and other aspects. In this study, the frequency band (0.0015–0.5 Hz) was selected for EGG signals to more accurately reflect the low gastric waves, which are typically at ~0.05 Hz or 3 cycles per minute (CPM). This ultra‐low frequency band provides an easy way to separate the basal electrical activity of the stomach, while allowing high‐frequency noise and very slow drift components. The results of pre‐processing are illustrated in Figure 3a. To improve the discrimination of these small bio‐signals, an amplified gain of 5000 was applied to make the low‐amplitude gastric waves appear more clearly, and thus more readily available for subsequent analysis. This filtering and amplification process produced a clean and biologically meaningful signal for the extraction of features of gastric functional behavior, including its transition from pre‐prandial to post‐prandial state.

### 2.5 Daydream Labeling Using EMG‐Based Physiological Trigger

**FIGURE 4:**
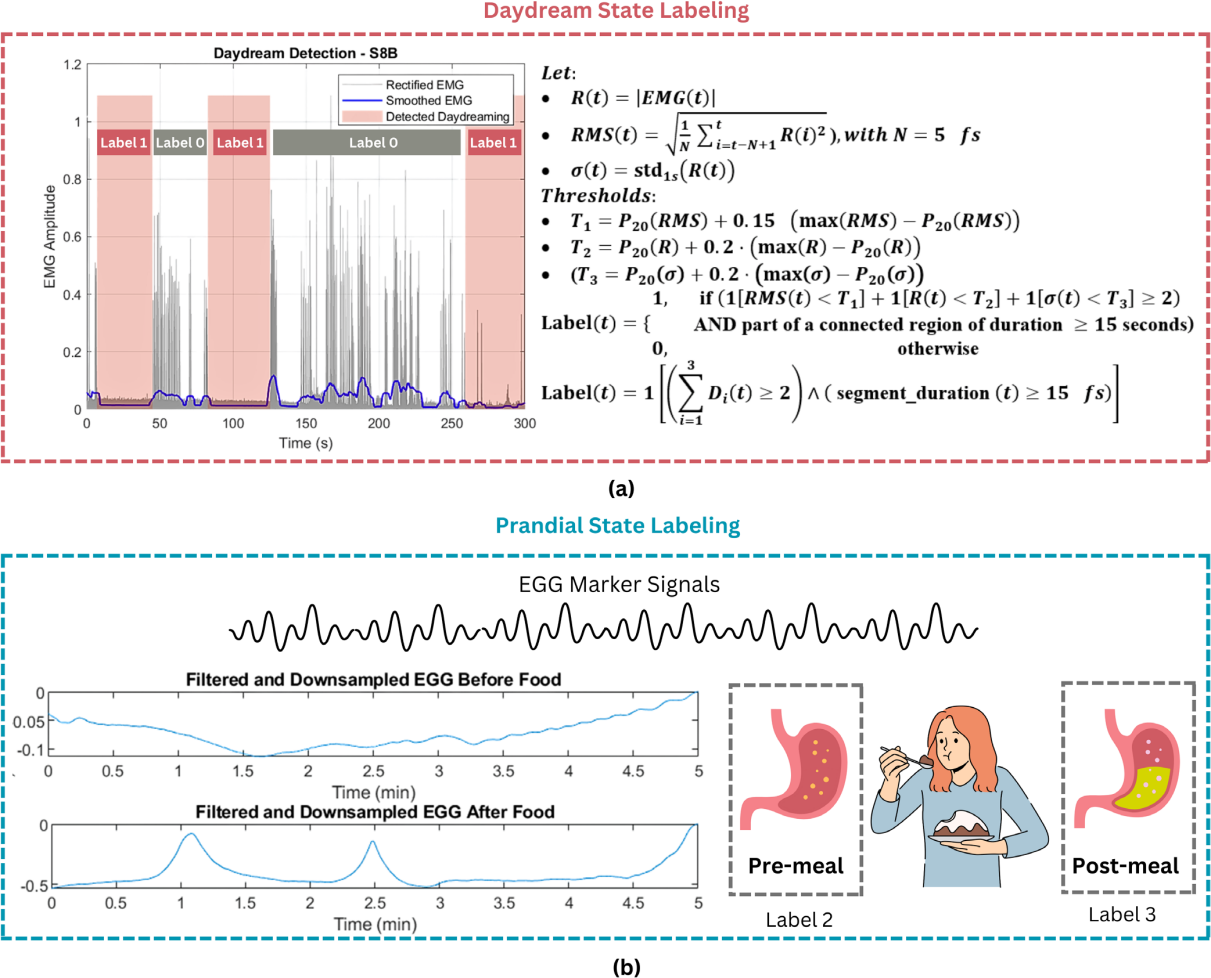
Daydream labeling algorithm design: (a) Thresholding on the basis of the three detectors, the rectified EMG, smoothed EMG (RMS), and standard deviation (std). The thresholds are defined with respect to the twentieth percentile of respective EMG functions. Labels, 1 and 0, are assigned as per detection. (b) Prandial State labeling depends on the files if they contain data of subjects in the pre‐prandial or post‐prandial state.

To detect the daydream segments, focus was on the low levels of muscle activity; these epochs reflect low EMG amplitude detected using surface EMG. It considers the three components of the EMG signal, the smoothed RMS, the raw rectified signal, and the moving standard deviation (SD) using the equation depicted in the Figure 4a. Each of the three representations has been useful in capturing some aspects of the underlying muscle activity [51, 52, 53, 54].

Three different thresholds for these signals are calculated from curves using a percentile method. Specifically, the 20th percentile of the signal was used as the baseline, and then a small amount of the range was added to the maximum value to produce a dynamic threshold. The EMG signal was below this value, likely indicating the user was DD. In addition, a voting method was also utilized, in that if two out of the three detectors simultaneously detected low activity, it indicated the signal was below threshold. This voting strategy prevented false detections, as shown in the graph of Figure 4a. The generic formula, depicted in Figure 4a, is represented by Label (t) where $D_{t}$ is linked with the thresholds as,

$$D_{1}\left(t\right)=1\left[\mathrm{RMS}\left(t\right)<T_{1}\right]$$

$$D_{2}\left(t\right)=1\left[R\left(t\right)<T_{2}\right]$$

$$D_{3}\left(t\right)=1\left[\sigma \left(t\right)<T_{3}\right]$$

Target was attained by smoothing (ignoring short and low‐activity breaks) and by only retaining episodes of more than 15 s as relevant daydream segments [55, 56]. This approach prevented false labeling from a short activity drop or noise. More generally, it has been used as a generic and adaptive method to detect periods of long‐lasting low muscle activity representing a disengaged or DD state. As a result, each DD segment was assigned label 1, whereas the other samples were labeled as 0.

### 2.6 Prandial State Labeling Using EGG Information

Regarding the prandial state identification, electrogastrography (EGG) data were collected in fasting state and fed conditions, as shown in Figure 4b. Each recording set was associated with the corresponding physiological condition on the basis of the time of the meal intake. Labels were assigned as label 2 for the entire EGG data of the pre‐prandial state and label 3 for the post‐prandial state. Such labeled files enabled the use of supervised machine learning for classification tasks that also trialed the characterization of the gastric activity pattern with respect to fasting and fed states.

### 2.7 Channel Selection of the fNIRS Signals

**FIGURE 5:**
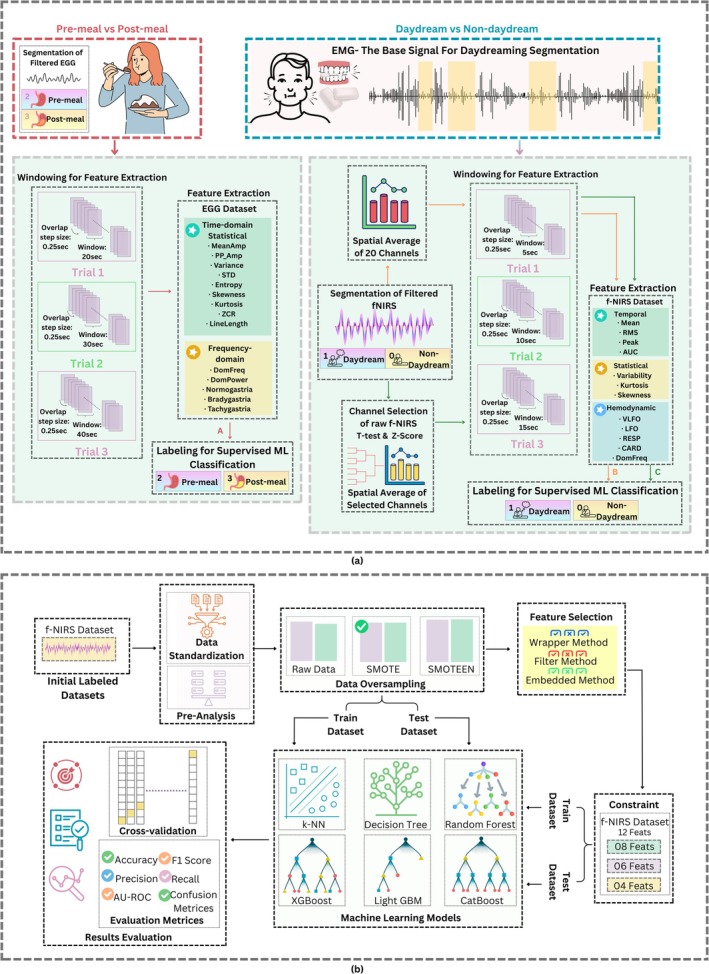
Complete Flow of Feature extraction and Classification: (a) Trial‐based windowing is performed on EGG and fNIRS signals, channels are selected for fNIRS signals, respective features are extracted, labeled files are ready to be fed into the ML classifiers; (b) labeled datasets are passed through the blocks of data standardization, processing, feature selection and ML classifiers to get the evaluation scores for analysis.

EMG and EGG signals were recorded from a single channel, but fNIRS signals were acquired via a 20‐channel optodes network. Hence, the subsequent step was fNIRS channel selection, which was the most important part to identify the promising channels for the correct analysis and classification. Because of the complexity of fNIRS data (multiple source‐detector pairs and dynamic hemodynamic responses across brain areas), it was required to select the most relevant channels to lower the noise, processing complexity, and overfitting. Various methods have been developed for the selection of a channel, for example, correlation‐based [45, 50, 57, 58]. *T*‐test and Z‐score methods were applied in the present study, as outlined in the subsections below and the workflow depicted in Figure 5a.

#### 2.7.1 *T*‐Test and Backward Elimination Method

The fNIRS channels were selected by coupling the *T*‐test with backward elimination, which allowed us to identify the channels that were most effective for distinguishing the classes between experimental conditions. First, the *T*‐test compared mean HbO responses for the DD and non‐DD groups across all channels. Channels with *p*‐values below the set threshold (*p* < 0.05) were marked as significant. Subsequent backward elimination removed, at each iteration, channels failing to meet the significance criterion, continuing until channels achieving consistent significance (*p* < 0.05) across the tests remained. This dual strategy effectively minimized channel count, retaining only the most informative features and thereby lowering dimensionality and enhancing the model's generalization capability.

#### 2.7.2 Z‐Score Method

The Z‐score channel filtering pinpointed the fNIRS channels fluctuating most with the hemodynamics linked to the DD state. Initially, a dynamic hemodynamic response function (dHRF) was synthesized. A standard gamma function was taken as the canonical model, and it was convoluted with a collection of binary vectors, each marked by the onset times of the classified DD epochs. This yielded a personalized dHRF for each subject, preserving the canonical shape. For every fNIRS channel, the strongest lagged cross‐correlation peak with the subject‐specific dHRF was evaluated, providing a scalar measure of the fNIRS channel's alignment with the expected neural response.

Subsequently, a Z‐score normalization was applied across the entire matrix of peak cross‐correlation strengths, centering the values on the subject‐wise mean and scaling by the standard deviation. Channels showing *z*‐values greater than zero, the ones with above‐average association to the dHRF, were retained, signaling enhanced coupling to the cognitive‐induced hemodynamics. The discarded channels, conversely, exhibited low task‐specific fluctuations.

### 2.8 Feature Extraction of the Physiological Signals

Once the relevant channels were chosen, the preprocessing stage involved feature extraction from the physiological signals—EGG and fNIRS—and was carried out entirely in MATLAB. The workflow aimed to transform raw time‐series samples into discriminative feature values to distinguish DD activity from non‐DD and the physiological state of pre‐meal versus post‐meal. The feature extraction served as the input to the classifiers trained during the subsequent stage, as shown in the Figure 5a.

A windowing constraint was applied during feature extraction to ensure both temporal coherence and physiological probability across the recorded signals of fNIRS and EGG. Because episodes of DD were constrained to a minimum length of 15 s, a moving‐window strategy was adopted for extraction. The windows were shifted by 0.25‐s increments, alternating between multiple fixed lengths: 5‐, 10‐, and 15‐s windows for fNIRS, and 10‐, 20‐, and 30‐s windows for EEG. These configurations are illustrated in the Figure 5a.

#### 2.8.1 Multi‐Domain Features Extraction of EGG Signals

After a typical meal, the electrogastrogram reflected a rise in CPM, as illustrated in Figure 4b, showing the increased gastric motility and active digestion. This elevation in CPM stems from the electrical activity of the gastric muscles, which become more excitable post‐ingestion, producing a series of slow waves that enhance both mechanical digestion and the forward movement of the meal. This physiological response of the stomach is quantifiable through EGG time‐ and frequency‐domain metrics.

The observed EGG pattern is quantitatively represented in the attached graph in Figure 4b, where time is on the horizontal axis and the corresponding pre‐ and post‐prandial CPM curve on the vertical axis. This ensures the role of EGG‐derived time and frequency metrics in monitoring food‐related physiological processes and provides confidence that analysis of pre‐ versus post‐prandial EGG records effectively characterizes meal‐induced gastric signal fluctuations.

In this study, 14 distinct EGG features were extracted, as summarized in Figure 5a. Overall, these features collectively address the temporal and spectral properties of gastric electrical activity. From the time domain, line length, variance, entropy, skewness, mean amplitude (Mean Amp), peak‐to‐peak amplitude (PP_Amp), standard deviation (STD), zero‐crossing rate (ZCR), and kurtosis are used to capture the periodicity, intensity, and structural complexity of the gastric signal. In parallel, the frequency domain measures—relative power in the normogastric band (Normogastria), tachygastric band (Tachygastria), bradygastric band (Bradygastrea), dominant frequency (DomFreq), and dominant power (DomPower).

#### 2.8.2 Multi‐Domain Feature Extraction of fNIRS Signals

A set of 12 fNIRS features was extracted and is summarized in Figure 5a. Given the inherently slower hemodynamic response observed in fNIRS, a minimum time window of 10–15 s is typically required to retrieve the complete shape of the oxy and deoxy‐Hb curves that correlate with cerebral activity [59, 60].

Absolute hemodynamic features that were extracted include mean, root‐mean‐square (RMS), peak, area under the curve (AUC), variability, skewness, and kurtosis, all of which quantify oscillations in blood oxygen content in a definitive manner. In parallel, frequency‐based features targeting very‐low‐frequency (VLFO), low‐frequency (LFO), cardiovascular band (CARD), respiratory band (RESP), and dominant frequency (DomFreq) [61] examine the oscillatory architectures that accompany neurovascular coupling, attentional reallocation, and the maintenance of cognitive state. Collectively, these multidimensional features served to decode the episodes of DD.

### 2.9 Feature Selection of fNIRS

In the pipeline of the fNIRS framework, following the feature extraction step [62], the most discriminative features that were able to differentiate DD states were selected as represented in the corresponding Figure 5b. Widely used feature selection techniques were incorporated for this purpose. Specifically, temporal and hemodynamic features deriving from frontal cortex activity were then filtered through the mutual information method [63, 64] and the recursive feature elimination approach [63]. The classifiers were experimentally validated, reporting the accuracy, precision, recall, and the F1‐score.

Three different complementary methods for feature selection were used in total: Mutual Information (MI), Recursive Feature Elimination (RFE), and Tree‐Based (TB) Feature Importance through the Random Forest model, which extracted the most predictive features from fNIRS data in the assessment of classification problems. The MI filter method measured the dependence of each feature on the target class, which reduced the redundancy and maximized the predictive power. Parallel to this, RFE functioned as a wrapper technique that employed a classifier to assign importance scores, recursively removing the weakest features until the desired set of smallest task‐specific features was reached. Additionally, TB rankings, an embedded approach, determined each feature's importance by calculating its average effect on the impurity reduction of a large ensemble of decision trees.

Across all techniques, subsets of 8, 6, and 4 features were examined, with subsequent performance of the classifiers measured using accuracy, precision, recall, and F1‐score. The study of three independent filter, wrapper, and embedded analyses facilitated a cross‐corner verification of performance metrics, revealing balanced minima against the feature set.

### 2.10 Four‐Class Labeling: Hybrid Dataset Labeling (LPT)

To relate DD to physiological shifts before and after meal intake, the four distinct behavioral classes guided by a powerset transformation [65, 66, 67] were identified. Initially, surface EMG dynamics were utilized to filter DD from non‐DD intervals. The outcome, a binary class, fills the primary label column, where “0” marks non‐DD and “1” marks an intrusively active state (DD). To embed the knowledge of prandial states in the workflow, a second label column followed. Each data file was tagged as “2” for pre‐meal or “3” for post‐meal according to whether the chunk was recorded during the 15 min immediately before or the 15 min after a feeding episode.

**FIGURE 6:**
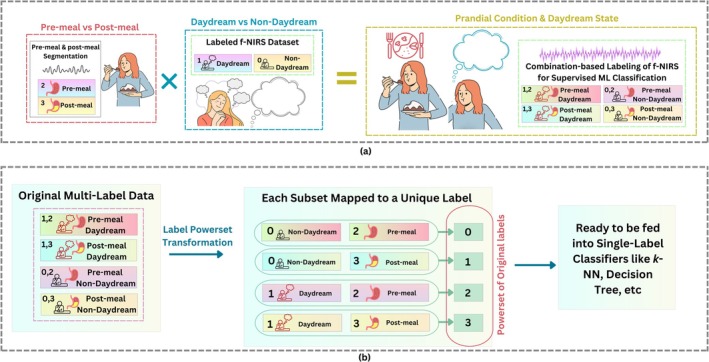
Preparation of hybrid datasets: (a) Combining the pre‐meal v/s post‐meal binary classes and DD v/s non‐DD binary classes to get the four‐class problem; (b) Implementation of Label Powerset Transformation (LPT) algorithm to transform four subsets of binary combinations into four individual classes.

The transformation followed in Figure 6a merges the two label columns using label powerset transformation, yielding four unique classes: (0,2) symbols for non‐DD pre‐meal, (0,3) non‐DD post‐meal, (1,2) DD pre‐meal, and (1,3) DD post‐meal.

The broad sequence for this approach is illustrated in Figure 6b. These labels set the stage for a four‐class classifier where the real objective was not merely to identify DD but to detect subtle shifts in cognitive mode linked with the timing of food intake.

### 2.11 Machine Learning Classifiers

Following feature selection, supervised classification was performed, creating both 2‐class and 4‐class datasets. The 2‐class problem aimed to distinguish DD from non‐DD periods, whereas the 4‐class problem included an additional dimension capturing cognitive state alongside whether the subject was in a pre‐prandial condition or not. A set of classifiers was tested: K‐Nearest Neighbors (*k*‐NN), Decision Tree, Random Forest, Extreme Gradient Boosting (XGBoost), Light Gradient Boosting (LightGBM), and Categorical Boosting (CatBoost). All models were trained on the selected feature subset using the train/test ratio of 70/30, and performance was assessed through accuracy, precision, recall, and F1‐score, as illustrated in Figure 5b. This classification step validated that DD produces rapid changes in feature values that are observable in association with the underlying physiological state, such as food intake.

## 3 Results

The datasets were organized to perform statistical analysis and to test both binary and four‐class classification problems. This allowed comparative assessments of the classification models for daydream monitoring in the pre‐ and post‐prandial state.

**FIGURE 7:**
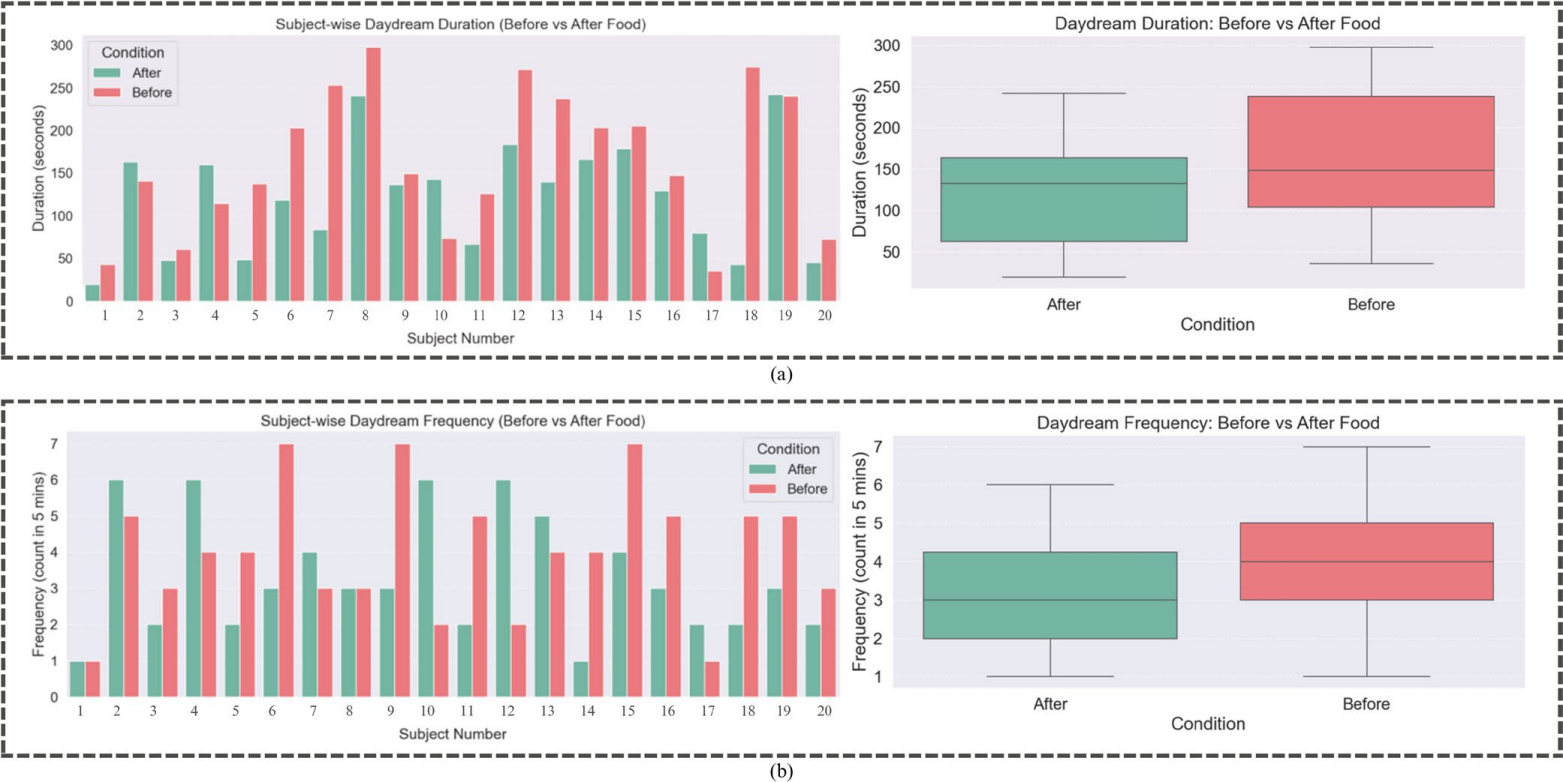
Daydream analysis with respect to prandial states: (a) daydream duration in pre‐meal v/s post‐meal samples; (b) daydream frequency in pre‐meal v/s post‐meal samples.

The bar plot in Figure 7a presents this statistical analysis. Each point on the horizontal axis represents a different subject, whereas the vertical axis sums daydream time in seconds during a narrowed five‐minute window, where episodes stay close to a cumulative peak of 280 s. Across participants, the data clearly show an increase in DD before eating and a symmetrical, pronounced drop soon after, coupled with an orderly trimming of episode durations among most individuals. This distribution makes it obvious that the pre‐meal mind offers a kind of receptivity to idleness of thoughts.

Finally, with respect to these findings, the box plot summary of DD time and subsequent count of DD episodes shows a downward (descending) trend, illustrated in the right plot of Figure 7a. On average, the overall length of these DD episodes decreased by 30%.

### 3.1 Daydream Duration in Pre‐Meal and Post‐Meal State

Daydream patterns monitored just before and right after meals reveal that participants' spontaneous DD episodes tended to last longer before meal intake, as illustrated in the Figure 7a.

### 3.2 Daydream Frequency in Pre‐Meal and Post‐Meal State

Similar patterns appeared in the frequency of both pre‐ and post‐meal daydream episodes, displayed in Figure 7b. For most participants, pre‐meal episodes occurred more frequently, showing a greater probability for spontaneous, off‐task thinking. In post‐prandial, a widespread dip in daydream frequency is observed for most of the participants. Although the absolute episode counts and individual slopes vary, a uniform direction emerges where DD becomes notably less frequent following the consumption of food. This effect is numerically tabulated using the box plot in Figure 7b, which indicates that on average, daydream episodes decrease by one‐third or 25% after eating.

#### 3.2.1 Effect of Food Coma on DD

In the present study, duration and frequency of DD in the post‐prandial state were quite low, paralleling the “low‐cognitive” state that a subject experiences immediately after lunch, which is often called “food coma” [68, 69]. Eating a full meal initiates a series of well‐known metabolic and hormonal responses, like insulin, additional serotonin (the calm‐producing neurotransmitter) releases, and the parasympathetic nervous system turns down from attentive to a calmer, less attentive state. The changes in non‐invasive fNIRS and EGG signals have been reflected in real‐time, so that the body's apparent postprandial shift is immediately transforming the DD patterns.

### 3.3 Hemodynamic Response and Brain Activation Maps

**FIGURE 8:**
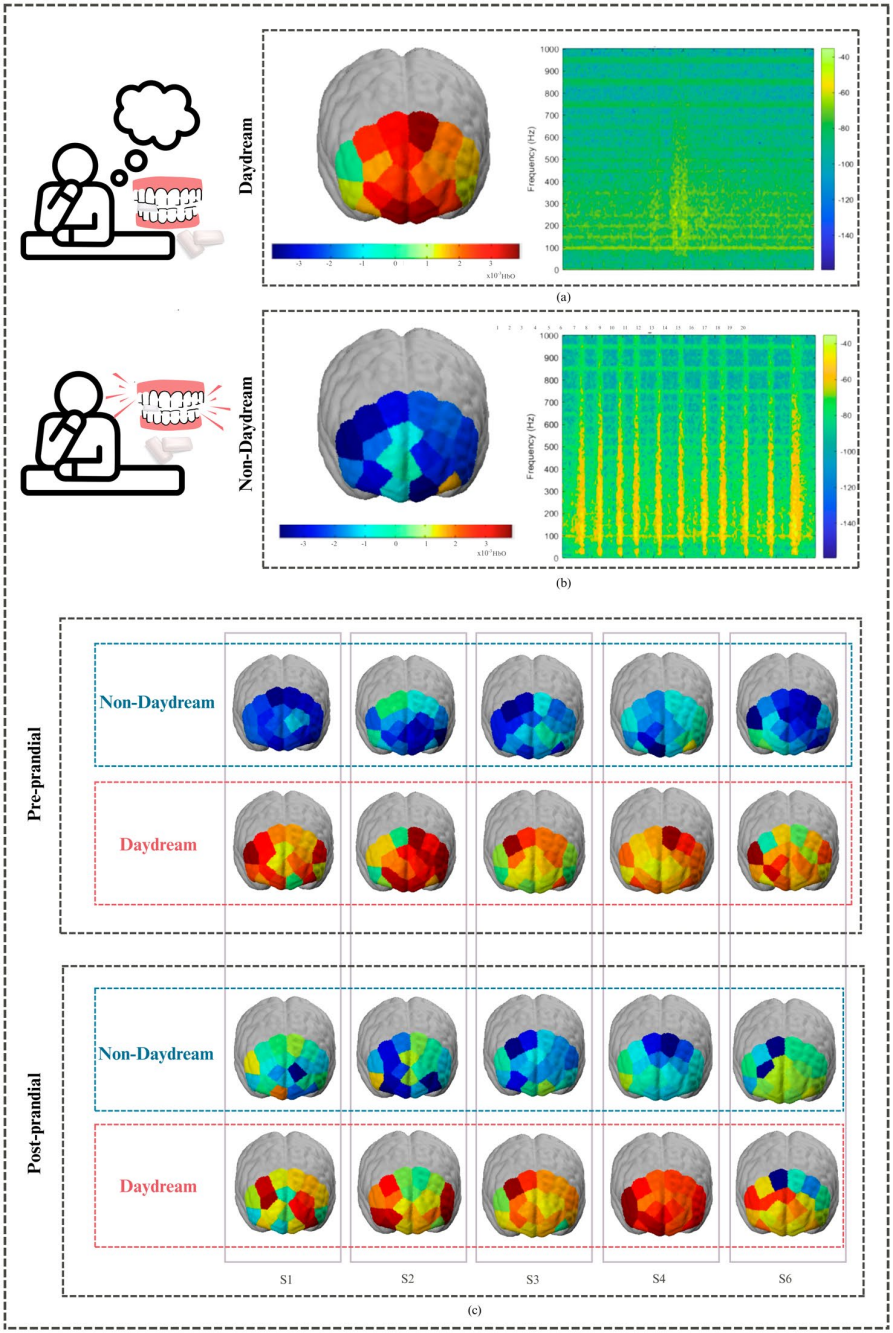
Brain activation maps: (a) Hemodynamic plot and EMG spectrograph during DD; (b) hemodynamic plot and EMG spectrograph during non‐DD; (c) comparative visualization of neuro‐hemodynamics during daydream and non‐daydream in pre‐prandial and post‐prandial states.

The fNIRS maps graphically depict the changes of cortical hemodynamic signals in terms of concentrations of HbO. Here, in Figure 8c, such maps are applied to direct comparisons of cognitive activation state within the same subjects, contrasting brain responses during brief episodes of spontaneous DD versus non‐DD in both the pre‐meal and post‐meal states.

Each DD episode is preceded and followed by brief non‐DD segments. In the corresponding spectrogram of the daydream state, in Figure 8a, The EMG yields only infrequent yellow peaks that predict mild muscular relaxation. On the other hand, the fNIRS cortical maps for the daydream state are dominated by vast orange patches, reflecting up to 3 × 10^−3^ HbO, extending over frontal and medial‐temporal cortex; these patches signal a synchronized rise in HbO indicative of marked metabolic demand during DD. Hence, activation peaks are observed within the medial and lateral prefrontal lobes, areas linked to vivid internal generation of imagination and the retrieval of relevant memories.

Conversely, fNIRS activation maps during non‐DD conditions display a wider expanse of blue shades, pointing to diminished cortical engagement, depicted in Figure 8b. Although the EMG exhibits well‐defined yellow peaks during the non‐DD segment depicted in the spectrograph. This is because the subject focuses more on chewing activity, enhancing the EMG amplitude. The darker blue bands in fNIRS signify a decreased percentage of HbO, indicating a decrease in regional cerebral blood flow as compared to periods of active DD. The largest drop in activation is observed within frontal and midline segments that align with the default mode network, reflecting a transition to externally focused attention, such as chewing.

Mapping brain activation before and after a meal while participants daydream reveals a marked post‐prandial decrease in neural activity, shown in Figure 8c. Plots taken before eating show elevated regions of activation, implying greater cognitive readiness; the post‐meal brain maps, however, present a pronounced drop, linked with an overall shift to a less alert state of food coma after meal intake.

In Figure 8c the subjects ($S_{x}$) are taken on the *x*‐axis, where *x* ranges from 1 to 5 five random subjects, and the *y*‐axis shows the contrasting brain maps of DD and non‐DD segments, during pre‐meal and post‐meal states. This subject‐wise analysis, contrasting the same individuals in both intervals, confirms a uniform pattern in which SiDD is observed more before meal intake, thus reaffirming well‐documented associations between food intake and transient cognitive decline. However, in rare cases, like one out of five subjects, the brain activation pattern of S4 showed deeper red regions depicting more intense DD after meal intake. This might be linked with the individual metabolic or psychological differences.

### 3.4 Window‐Based Results: Two‐Class Daydream Classification With fNIRS

**FIGURE 9:**
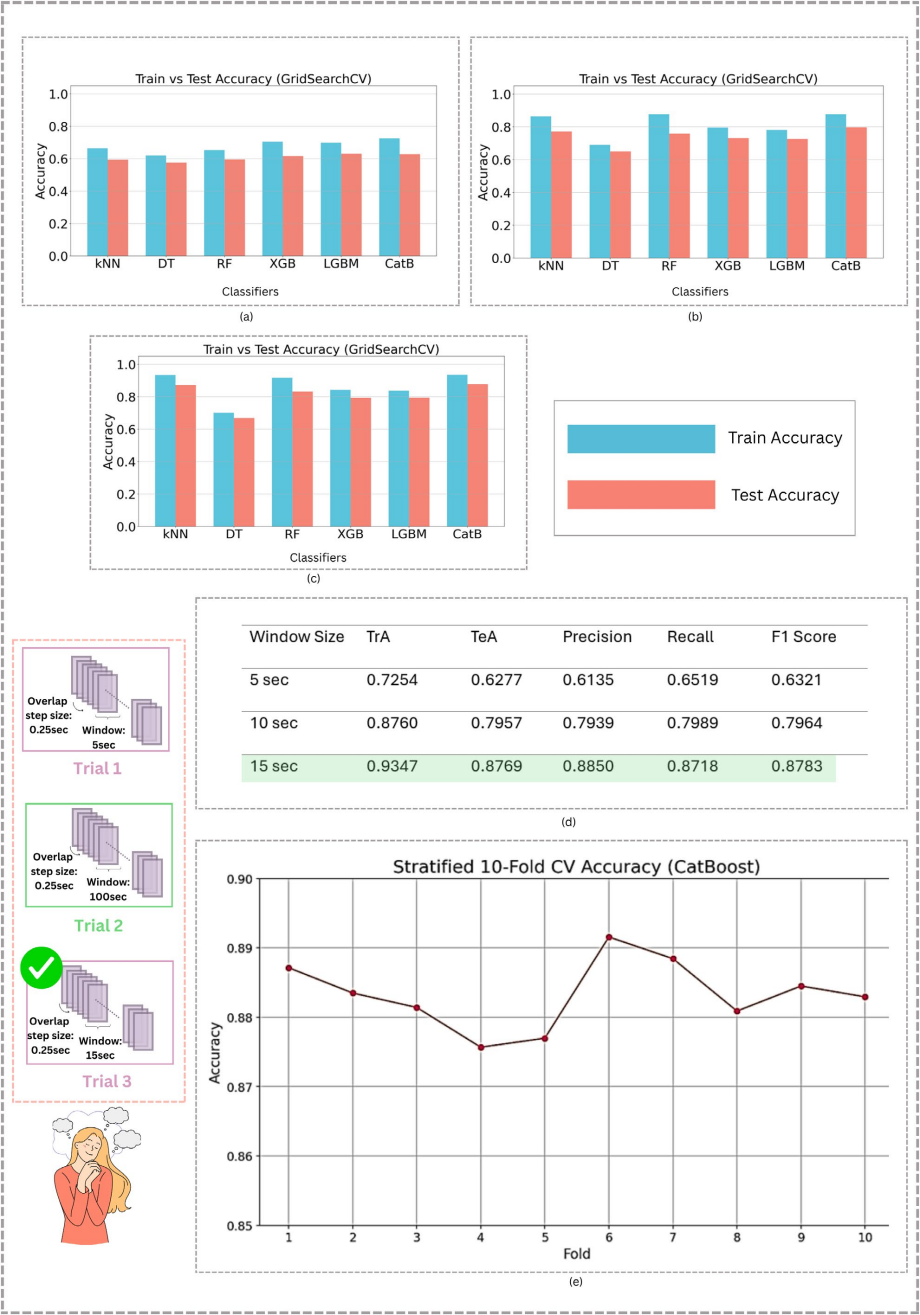
Trial‐based windowing analysis: (a) FNIRS classification accuracy for the feature set extracted on 5 s windows; (b) FNIRS classification accuracy for the feature set extracted on 10 s windows; (c) FNIRS classification accuracy for the feature set extracted on 15 s windows; (d) Evaluation scores for the feature set extracted on 15 s windows; (e) 10‐fold cross‐validation for the fNIRS dataset.

Across three temporal windows of fNIRS, 5 s, 10 s, and 15 s, the outcomes for the fNIRS signals clearly show a progressive gain in the classifier's performance as the window duration increases, shown in Figure 9a–c, respectively. Each plot reports the training and test accuracy for all three classifiers, confirming that CatBoost leads in every evaluated duration. At the 5‐s window, shown in Figure 9a, all classifiers yield similar, low accuracies. When the feature extraction window expands to 10 (referring to Figure 9b) and subsequently to 15 s (referring to Figure 9c), the results are clearly enhanced, with CatBoost showing correspondingly superior test accuracy. This consistency implies that the algorithm reliably learns intricate patterns in the underlying hemodynamic response, asserting its robustness in processing fNIRS time‐series data.

The trends depicted in Figure 9d clearly show that enlarging the feature extraction window yields noticeably better accuracy. A 5‐s window records a test accuracy of 62.77% paired with an F1 score of 63.21%, since that interval does not sufficiently characterize the task‐related hemodynamic shift. Accuracy rises to 79.57% with a 10‐s window, then to 87.69% with a 15‐s window, whereas the same pattern is observed in precision, recall, and F1. Every performance score indicates that a longer duration allows the classifier to better recognize the gradual hemodynamic shifts that characterize functional near‐infrared spectroscopy.

fNIRS signals are based on slow, level‐shifting hemodynamic responses, with a peak occurring a few seconds after a stimulus. A wider window interval captures the complete HbO and HbR changes, recording both the ascent and descent and thus yielding superior classification scores.

Moreover, these findings were validated with a 10‐fold cross‐validation procedure, illustrated in Figure 9e. Other classifiers were also used for 10‐fold cross‐validation, as depicted in Figure S1a–f of the Supporting Information. Across all folds, performance metrics remained stable within a 15‐s time window. The observed variance was minimal, indicating that the fNIRS classifier architectures are insensitive to the data sub‐groupings.

### 3.5 Channel Selection of the fNIRS Dataset

Afterwards, the fNIRS channel selection was performed to identify the optimally informative channels, those that showed the strongest activation patterns while participants underwent DD. This channel filtering step simultaneously reduced the computational load and enhanced model sensitivity and specificity.

#### 3.5.1 Results of *T*‐Test and Backward Elimination

To evaluate performance across different fNIRS channel sets, backward elimination, along with paired *t*‐tests, was employed. In contrast, all 20 channels yielded the training and testing accuracy of 93.47% and 87.69%, coupled with an F1 score of 87.83%, as presented in Table 2. Interestingly, the model exhibited improved performance with channel count reduction. Configurations utilizing at most 5 channels or at most 15 channels recorded a modest uplift, with an F1 score of 88.77; a maximal channel count of 10 achieved balanced metrics, producing a testing accuracy of 89.09% and an F1 score of 88.96%.

**TABLE 2:** Classifier Evaluation Scores of CatBoost for Channel Selection via *T*‐test and backward elimination, including all channels, less than or equal to (≤) 5 channels (5CH), 10 channels (10CH), 15 channels (15CH), and Auto selection method (Auto CH). Scores include Train Accuracy (TrA), Test Accuracy (TeA), Precision, Recall, and F1 score.

Channels	TrA	TeA	Precision	Recall	F1 score
All channels	0.9347	0.8769	0.8850	0.8718	0.8783
≤ 5CH	0.9571	0.8820	0.8831	0.8923	0.8877
≤ 10CH	0.8896	0.8909	0.8989	0.8949	0.8896
≤ 15CH	0.9571	0.8820	0.8831	0.8923	0.8877
Auto CH	0.9673	0.9029	0.9063	0.9081	0.9072

*Note:* Highest test accuracies are highlighted with gray shade.

The auto channel selection method, which means different numbers of channels across different subjects, showed the best results with training and testing accuracies of 96.73% and 90.29% alongside precise performance measures such as precision, recall, and F1 score averaging 90.63%, 90.81%, and 90.72%, respectively. Collectively, these results prove that smaller feature sets, when properly optimized, exceeded detection accuracy previously obtainable only with the 20 channels, thereby enhancing computation efficiency while improving classification results.

#### 3.5.2 Results of Z‐Score Channel Selection

The z‐score‐based channel selection method was also integrated to enhance the fNIRS system for daydream‐state identification, focusing exclusively on the most statistically important channels. The original model, utilizing the 20‐channel array, yielded a training accuracy of 93.47%, a test accuracy of 87.69%, and an F1 score of 87.83%, all recorded as baseline performance metrics.

Subsequent reduction to five or fewer channels, filtered by Z‐score thresholds, yielded a test accuracy of 89.20% and achieved an F1 score of 89.39%, as detailed in Table 3.

**TABLE 3:** Classifier Evaluation Scores of CatBoost for Channel Selection via Z‐score, including all channels, less than or equal to (≤) 5 channels (5CH), 10 channels (10CH), 15 channels (15CH), and Auto selection method (Auto CH). Scores include Train Accuracy (TrA), Test Accuracy (TeA), Precision, Recall, and F1 score.

Channels	TrA	TeA	Precision	Recall	F1 score
All Channels	0.9347	0.8769	0.8850	0.8718	0.8783
≤ 5CH	0.9585	0.8920	0.8862	0.9016	0.8939
≤ 10CH	0.9567	0.8819	0.8775	0.8903	0.8839
≤ 15CH	0.9556	0.8841	0.8780	0.8946	0.8862
Auto CH	0.9568	0.8838	0.8714	0.9030	0.8869

*Note:* Highest test accuracies are highlighted with gray shade.

Experiments with different channel configurations, which limit channels to 10 and 15, achieved marginal degradation in terms of performance, with the precision, recall, and F1 measures around 88% via the CatBoost classifier. This regularity also explains how the Z‐score method generalizes well across channel numbers. Similarly, the Z‐score selection‐driven configuration resulted in 89.20% test accuracy and a similar F1 score of 89.39% with the threshold of 5 channels. Taken together, these metrics confirm that the Z‐score guided channel pruning for dimensionality reduction consistently removes channels and enhances classification performance, performing its part in refining fNIRS signals for cognitive‐state tasks.

In comparison with the results, the *t*‐test method on the basis of the auto‐selected channel set, after backward elimination, was better in performance than the Z‐score condition, with a superiority of 2% in accuracy, and achieved an F1 score of 90.72%. This is consistent with the threshold‐based variable selection; the *t*‐test method carried out in processing specific channels that were maximally involved in the detection of DD in the subjects. More generally, the statistical channel approach reduced the dimensionality of the signals without adding performance‐degrading discretization.

### 3.6 Feature Selection of fNIRS Data

Each feature selection technique, Mutual Information (MI), Recursive Feature Elimination (RFE), and Tree‐based (TB) method, yielded a distinct outcome when tested on the fNIRS physiological signals, summarized in Table 4. Mutual Information, when restricted to eight features, selected Peak, Root Mean Square, VLFO, Variability, Mean, CARD, DomFreq, and Skewness. This selection mixed classical statistical and physiological features, indicating a preference for features closely linked with the class‐label purity. RFE, in contrast, selected the first 10 features that were Mean, RMS, Peak, AUC, Variability, Kurtosis, Skewness, VLFO, and LFO.

**TABLE 4:** Results analysis of FNIRS feature selection showing evaluation scores of CatBoost Classifier for: All 12 features, 8 features (Best results obtained via Tree‐Based method), 6 features (best results obtained via recursive feature elimination), and 4 features (Best results obtained via Tree‐Based method). Scores include Train Accuracy (TrA), Test Accuracy (TeA), Precision, Recall, and F1 score.

No. of features	TrA	TeA	Precision	Recall	F1 score
12 (All)	0.9673	0.9029	0.9063	0.9081	0.9072
8 (TB)	0.9604	0.9077	0.9196	0.9023	0.9109
6 (RFE)	0.9475	0.8976	0.9076	0.8952	0.9014
4 (TB)	0.9156	0.8725	0.8791	0.8767	0.8779

*Note:* Highest test accuracies are highlighted with gray shade.

When the feature set was reduced to six variables, Mutual Information selected Peak, RMS, VLFO, Variability, Mean, and CARD, while dismissing DomFreq and Skewness. RFE limited the same subset to Mean, RMS, Peak, Variability, Kurtosis, and Skewness. The Tree‐based algorithm, conversely, selected Peak, VLFO, RMS, LFO, Skewness, and Kurtosis. At the threshold of the top 4 features, Mutual Information selected Peak, RMS, VLFO, and Variability. Recursive Feature Elimination trimmed the feature set to Mean, RMS, Peak, and Skewness, revealing the dual significance of variability and low‐frequency behavior. The tree‐based method retained Peak, VLFO, RMS, and LFO, further underscoring the dominance of non‐linear and dynamic variability features, even in a limited set. Collectively, the three approaches, MI, RFE, and the TB method, mutually selected Peak, RMS, and VLFO as common features, yet each shifted its rank of feature importance according to the method's architecture.

The dataset underwent classification using a CatBoost classifier, and one of the three feature selection techniques that gave the highest train and test accuracy was selected across each threshold, 8, 6, and 4 features, as documented in the Table 4. In the case of the threshold of top eight features, features selected by the tree‐based technique outperformed the datasets having features selected by the other two methods, resulting in a test accuracy of 90.77%.

Inspection of the retained features reveals that spectral variance components (VLFO and LFO) and simple statistical moments (Skewness and Kurtosis) dominate the selection, confirming that these metrics vary with the underlying physiological shifts that correspond to differing cognitive loads. In parallel, both RFE and MI scoring deliver relevant rankings but ultimately yield marginally lower results, like test accuracy of 89.76% with a six‐feature subset.

### 3.7 Prandial State Classification With EGG: A Two‐Class Problem

A 30‐s window was opted for when processing EGG data because gastric slow‐wave activity occurs at exceptionally lower frequencies, around 3 CPM. The 10‐ and 20‐s alternatives were also tested, yet these often terminated before a complete cycle could appear, restricting the computation of key features and rendering some of the time‐ and frequency‐domain metrics partially missing. Extending the duration to 30 s reliably extracted an integer number of cycles on almost all recordings, yielding an integer number for all features.

To discriminate between pre‐ and post‐meal states, all 16 electrogastrogram (EGG) features were utilized. The half‐minute recording frame was long enough to collect one full gastric cycle. Because nearly all slow‐wave features are sensitive to prandial shifts, this aggregated feature set served as a reliable criterion for detecting the physiological transition. Following feature extraction, classification accuracy was computed for each subject individually, employing traditional metrics such as accuracy, precision, etc.

**FIGURE 10:**
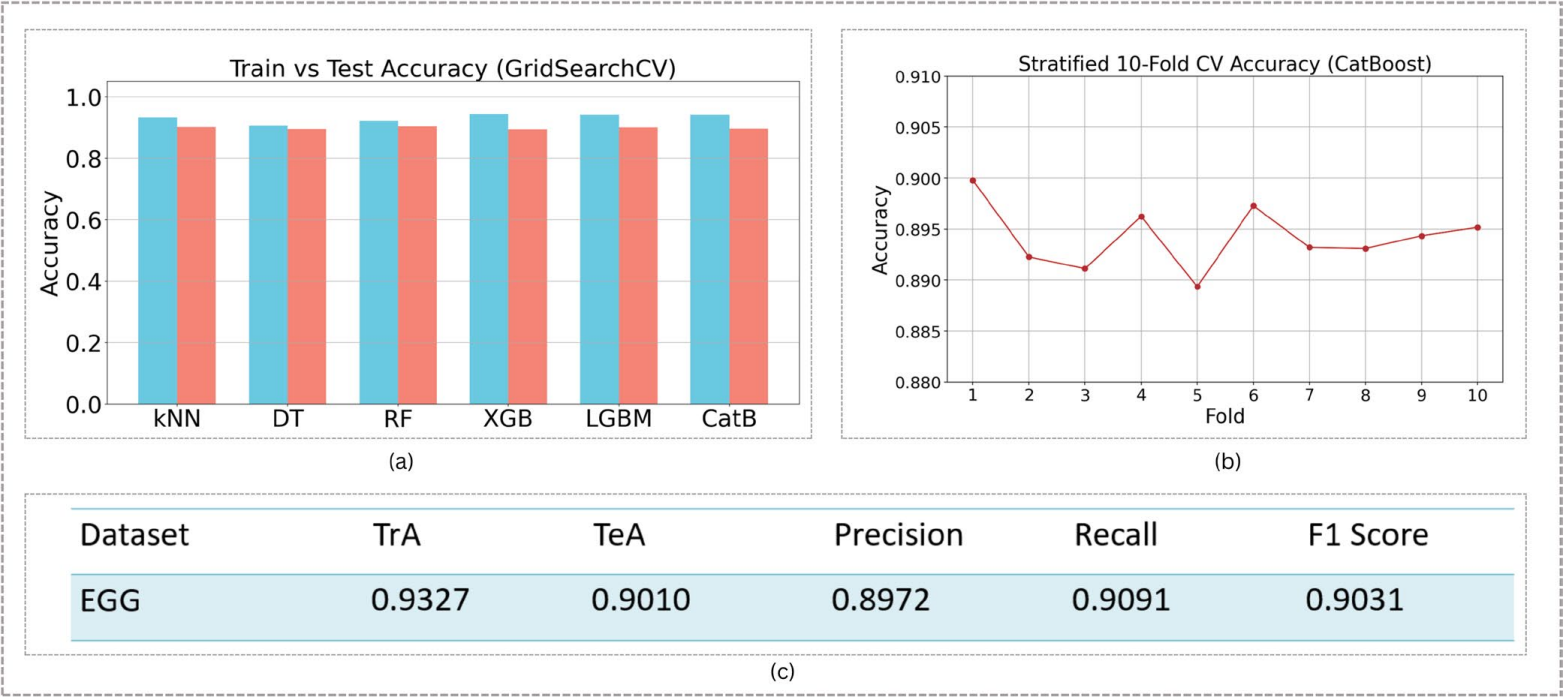
EGG Classification Analysis: (a) Train and Test Accuracies across all classifiers; (b) 10‐fold cross‐validation for CatBoost (best performer); (c) evaluation scores including Train Accuracy (TrA), Test Accuracy (TeA), Precision, Recall, and F1 score for CatBoost classifier.

Referring to Figure 10a,c, the classification results from the EGG‐based prandial state detection reveal uniformly strong performance across all tested classifiers, with minimal accuracy deviations among the different models. CatBoost clearly outperformed the others, delivering the best test accuracy of 90.1%. To strengthen these findings, a 10‐fold cross‐validation procedure was applied, as shown in Figure 10b, confirming that accuracy metrics remained stable across repeated folds and that variance among the fold results was acceptably low. Therefore, these findings reveal the excellent discriminative capacity of the EGG feature set and, additionally, justify the selection of a 30‐s sliding window for classifying the prandial states.

### 3.8 Prandial State Classification With fNIRS: A Two‐Class Problem

This study also incorporated fNIRS signals to classify pre‐prandial and post‐prandial gastric states by analyzing neural hemodynamic patterns. A two‐class supervised algorithm trained on feature sets derived from fNIRS time series attained a mean training accuracy of 95.78% and testing accuracy of 90.64%, confirming robust model stability across datasets. Further metric examination yielded precision at 90.29%, recall at 91.08%, and an F1‐score of 90.68%, evidencing both sensitivity and specificity in labeling pre‐ and post‐meal epochs without bias toward either class. Collectively, the strength of the extracted neuro‐vascular features corroborates the capability of fNIRS signals to detect mild and transitory post‐meal neuro‐physiological shifts, thus supporting the modality's deployment in real‐time prandial‐state recognition.

### 3.9 Four Class Classification Problem

The four‐class task to distinguish between DD and prandial states added another dimension to the architecture relative to the prior analysis, as illustrated in Figure 6a,b. Differentiation between cognitive and physiological variations was pursued in parallel streams.

**FIGURE 11:**
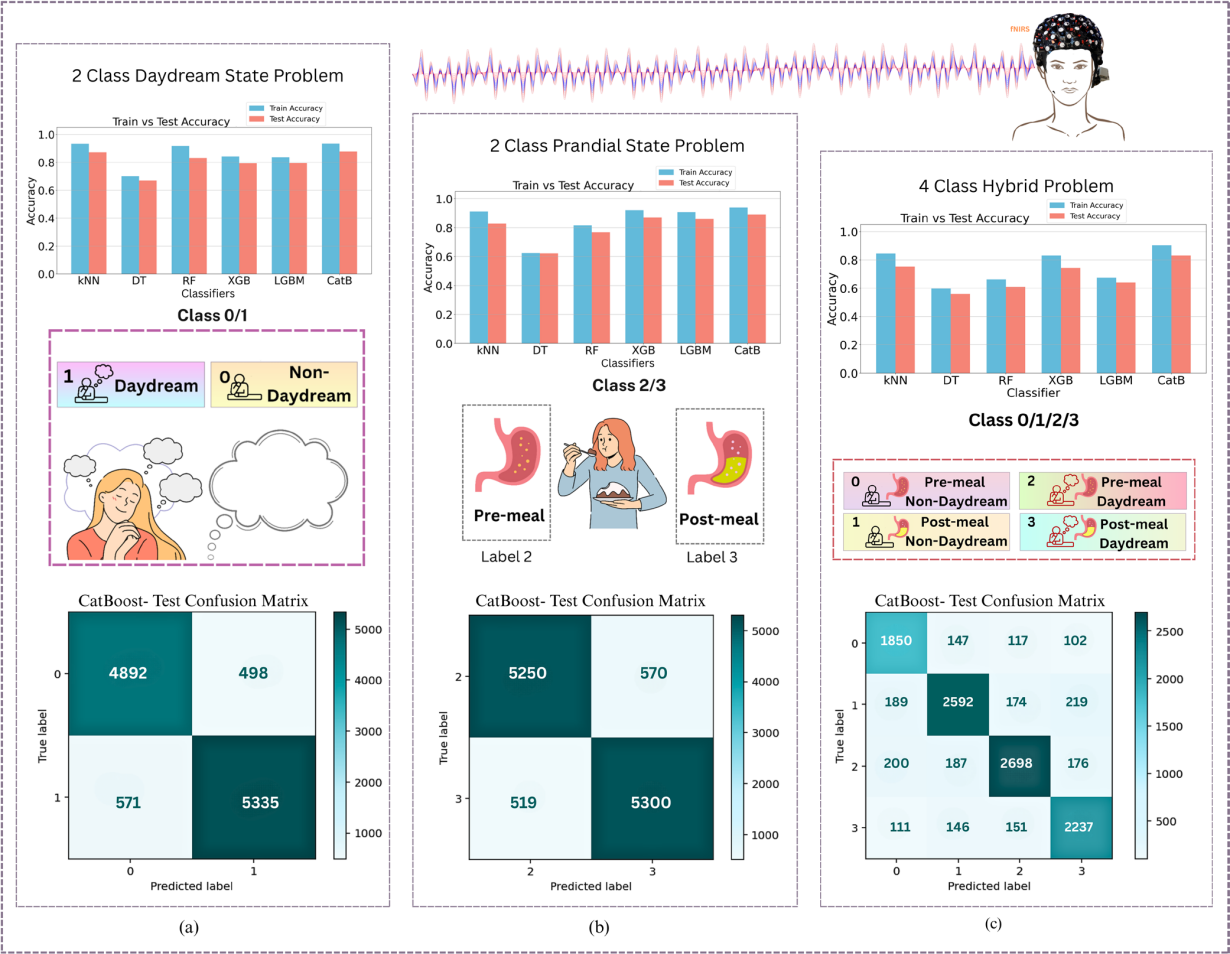
Comparative analysis of the two two‐class problems and a four‐class hybrid problem: (a) Classification results including the train v/s test accuracies and confusion matrix for two‐class daydream detection via fNIRS; (b) Classification results including the train v/s test accuracies and confusion matrix for two‐class prandial state detection via fNIRS; (c) classification results including the train v/s test accuracies and confusion matrix for four‐class daydream detection incorporating prandial state knowledge via fNIRS.

The fNIRS system did not yield as good a performance on the hybrid four‐class task as achieved in the two‐class problem. It resulted in the train accuracy of 90.44%, test accuracy 83.01%, precision 82.71%, recall 83.22%, and f1 score 82.88%. To interrogate the underlying causes, these findings were subjected to pairwise examination. Analysis depicted in Figure 11a–c illustrates that the performance bars for the fNIRS display consistent tendencies across the two experimental contexts, the two‐class daydream classification problem and the two‐class prandial state classification problem. In both separate binary problems, fNIRS achieved comparatively robust discrimination, leveraging the distinct hemodynamic characteristics that arise when the brain is either in a self‐induced DD state or a non‐DD state, or differing nutritional phases (pre‐prandial and post‐prandial).

When the two scenarios, cognitive and prandial, were integrated and all four classes were presented simultaneously, performance on the fNIRS metric dropped markedly (Test Accuracy: 83.01%, F1 Score: 90.44%). The attenuation in statistics indicates that the pertinent hemodynamic patterns are converging or losing distinctive features once the dimensionality increases. The interpretation is that the simultaneous assessment of mind‐wandering and metabolic state composes a composite source of variation beyond the encoding capacity of the fNIRS channels, or that may require different features to detect both activities simultaneously.

In the confusion metric of Figure 11a, for binary DD detection using fNIRS, the model shows strong performance with True Positive (TP) = 5335, True Negatives (TN) = 4892, False Positives (FP) = 498, and False Negatives (FN) = 571. The high TP and TN counts indicate that the classifier correctly distinguishes between DD and non‐DD states most of the time, with relatively few misclassifications. The FP and FN rates are low, suggesting balanced detection accuracy across both classes. In the confusion metric of Figure 11b, for binary prandial state detection, the confusion matrix similarly demonstrates high classification reliability, with TP = 5300, TN = 5250, FP = 570, and FN = 519. This close balance between FN and FP indicates that the model performs equally well across both prandial states. Like Figure 11a, there is strong predictive power, but slight misclassifications remain.

In contrast, in the confusion metric of Figure 11c, the 4‐class hybrid analysis introduces more complexity, resulting in some performance degradation. Although diagonal values (TPs per class: 1850, 2592, 2698, and 2237) are relatively high, the off‐diagonal counts show more frequent misclassifications. Here, each class has distributed FP and FN rates, reflecting that distinguishing between multiple states is more challenging than binary classification. Nevertheless, the overall diagonal dominance suggests that the model retains good discriminative ability across four classes.

## 4 Discussion and Future Recommendations

The findings of this study reveal that optimal machine learning, in combination with physiological signals, can detect DD in the prandial state in real time, providing insights into model performance and potential areas for further development.

This research introduced a physiological trigger‐based mechanism for detecting daydreams using EMG signals. It has been seen that EMG signals can serve as a biomarker in case of attention disengagement, and it was observed that the chewing activity was also naturally reduced when the subjects were in the state of DD, as shown in Figure 4a. Three detectors were, in total, employed: one with the rectified amplitude, one with smoothed RMS, and a third with the moving standard deviation of the recorded signal. The detection system determined periods of little activity as probable bursts of DD when at least two detectors agreed. This procedure was used to achieve non‐biased annotations across trials, as participants' data were logically labeled along with the subject's feedback. This labeling was highly valuable as it allowed us to prepare for AI‐based learning models on the basis of indicative physiological patterns of DD.

The statistical comparison of DD episodes presents a significant difference in daydream duration between subjects 8 (around 220–290 s) and 20 (around 30–60 s). It varies subject to subject as it depends on the natural cognitive tendency of the person to experience self‐induced DD, which is related to how easily and how frequently the person shifts their attention from the present environment to their internal thoughts. Moreover, it also varies person to person in how long they can maintain their state of self‐induced daydreaming. DD is a state‐related and extremely individualized cognitive phenomenon, not an event, and not time‐locked. The variation of baseline cognitive control, imagination trend, emotional involvement, and autonomic‐cortical physiology implies that some people indulge in DD with shorter episodes, whereas others have fewer or longer episodes. Hence, the observed variance is due to real neurophysiological differences as opposed to experimental anomaly and is a natural feature of real‐life DD behavior.

In this study, for DD classification on the basis of fNIRS signals, CatBoost resulted in the highest F1 Score of 90.72% with the CatBoost model, in contrast to the study [27], where fNIRS signals were utilized to classify the mind‐wandering state with the highest F1 score of 73% with the SVM model. Similarly, in reference to our interest in stomach states classification, the study performed by M. Faizal et al. [34] compared five features for separating pre‐prandial from postprandial states, where two classifier models were tested, SVM and ANN, and the highest classification accuracy of 82.3% was achieved by the SVM classifier. However, this study presents the highest test accuracy of 90.1%.

Moreover, the effect of meal intake on attentional regulation and cognitive state was supported by pre‐ and post‐prandial significant physiological differences. Several features, such as fNIRS HbO concentrations (observed in brain activation maps of Figure 8a–c), significantly differed during DD. More intense brain activation has been observed in pre‐meal plots. Moreover, following a meal, the duration and frequency of DD decreased (shown in Figure 7a,b), indicating the common feeling of “food coma” that is induced by post‐prandial conditions like metabolic and hormonal responses, such as increased insulin, serotonin release, and parasympathetic nervous system activity. Together, these physiological responses contribute to a less aroused, more relaxed state of mind. By making the recording in multiple trials using a non‐invasive fNIRS in combination with the EGG system, it has been made possible to capture real‐time objective evidence on how the body's postprandial changes directly impact cognitive activities. The present quantitative results further establish the association between digestion‐related physiological activities and decreased SiDD, and support that the spontaneous brain drift is significantly attenuated following food intake.

Also, the experiments presented in this paper indicate that with the window‐based approach, longer temporal windows were significantly better in extracting physiological features for daydream detection. Application of three windows lengths, 5‐, 10‐, and 15‐s, for fNIRS and 10‐, 20‐, and 30‐s for EGG, confirmed the benefit of longer windows, 15 s for fNIRS and 30 s for EGG, which better utilize the hemodynamic trend from fNIRS signals (depicted in Figure 9d) and physiological variations from EGG. Larger windows were more robust for extracting features, where the classifier performance was enhanced in the modalities to better ensure the reliability of the detection of transient cognitive states of daydream and prandial state from the electrical activity of the stomach. Afterwards, classification algorithms were employed for this problem, and out of the six classifiers, CatBoost achieved strong physiological separability when distinguishing between pre‐ and post‐meal states, with an accuracy that reached up to 90.11% on the basis of EGG data, as depicted in Figure 10c. Similarly, for fNIRS classification, CatBoost resulted in the highest accuracy of 87.69%.

Then, the Channel selection and dimensionality reduction methods were applied to the fNIRS 20‐channel data. According to a 15‐s sliding window dependent feature selection, the associated classification results also achieved an improvement on the classification performance with an increasing test accuracy from 87.69% to 90.29% (i.e., an increase of 2.6%) after channel selection and then 90.77% (further increase of 0.48%) after feature selection (referring to Tables 2 and 4). Looking into the Table, TB (tree‐based) selection ranks first. Since fNIRS was able to measure the large neural variation because of cognitive state on the basis of the oxygenation changes, the test results achieved an F1‐Score of 91.09% and a test accuracy of 90.77%. In general, Windows‐based analysis combined with the proper selection of features was very effective in improving the classification of all the modalities.

Auto Channel (CH) selection by the *T*‐test method was the best (referring to the results of Table 2) and the only method that allowed the automatic selection of one optimal subset of channels, whereas a fixed number of channels (e.g., 5, 10, or 15 channels) did not perform well over all subjects and gave a lower accuracy. Because of variable signal quality and differences in channel performance, the number of most informative channels varied among subjects. By adjusting channel selection on a per‐participant basis, the Auto CH approach made the most use of high‐quality discriminative channels and, therefore, achieved higher classification accuracy, which is shown in Table 2. Comparing the two methods, the Z‐score and *t*‐test (results depicted in Tables 2 and 3), backward elimination used with the *t*‐test was better than Z‐score by 1.09%, and it achieved an accuracy of 90.29% along with an F1 score of 90.72%. Whereas the Z score achieved a test accuracy of 89.20. This verifies the rank‐ordered channel selection by the *t*‐test approach, employing information‐specific channels, which best supported the detection of DD in the individuals.

In the present work, two binary label columns were combined, on the basis of the Label Powerset method, to generate a 4‐class label for joint analysis of DD and prandial states (referring to Figure 6b). Four classes corresponding to different physiological and cognitive states were created for each pair of labels [0, 1] in one column with [2, 3] in the other. This mechanism effectively combined the influences of both original labels to obtain a more condensed signal, which can be more easily learned and with more consistent features selected by the model. Nevertheless, the test accuracy decreased to 83% for the 4‐class problem; at the same time, the train–test accuracy gap increased.

The observation regarding hybrid analysis reveals that relying solely on unimodal fNIRS signals for classifying daydream and non‐daydream states, good results are achieved. To study events like mind‐wandering and prandial state simultaneously, where behavioral states overlap, a richer feature set might be required. To solve this, including richer features, such as temporal, spectral, and connectivity‐based features from fNIRS and EGG, may be able to better detect subtle physiological changes. Furthermore, investigating other multi‐label encoding techniques, for example, classifier chains, could potentially improve the model's performance and generalization.

## 5 Conclusion

This research proposes an AI‐integrated system to monitor the DD states on the basis of human physiological signals of fNIRS using an EMG‐based trigger. Additionally, the influence of prandial states on mind‐wandering behavior was also studied using EGG signals. Analysis of results showed that both the DD duration and DD frequency were significantly reduced by post‐meal intake, by around 25% and 30%, respectively, thus proving the powerful impact that postprandial physiological changes have on the cognitive activity of DD. For better performance of the ML classifiers, channel and feature selection strategies were used. In the case of fNIRS channel selection, the Auto CH method that utilizes the *T*‐test resulted in the improvement of test accuracy by about 2.6% (from 87.69% to 90.29%), and the tree‐based feature selection further enhanced an extra gain of 0.48%. CatBoost performed better among all six ML classifiers with the test accuracy of 90.77% for binary DD detection and 91% for prandial state classification. For a 4‐class hybrid classification, a maximum accuracy of 83% was achieved, suggesting that the multi‐class modeling could benefit from further improvements. Together, these results confirm that non‐invasive measurement techniques, the fNIRS and EGG recordings, can detect real‐time changes in cognitive and physiological state; thus, it is possible to distinguish DD states. Further work could be performed on complex multi‐label methods to enhance generalization. Moreover, lightweight ML models deployed on embedded hardware could support on‐device inference, real‐time feedback, and continuous monitoring for practical applications in cognitive health, behavioral research, and daydream monitoring.

## Author Contributions

Anusha Ishtiaq contributed to the study design, data collection, formal analysis, and manuscript writing; she also ensured the integrity of the data. Zia Mohy‐Ud‐Din provided technical expertise, guided the methodology, and critically reviewed the manuscript. Noman Naseer also helped with technical expertise and technical proofreading of the manuscript, and Abdullah Al Aishan assisted with funding acquisition and the final review of the draft. Jahan Zeb Gul contributed to the conceptualization, initial idea, research direction, and final draft review. Syed Ghufran Khalid contributed to the initial idea, result analysis, and data review, as well as the review of the final draft.

## Funding

The authors extend their appreciation to the Deanship of Research and Graduate Studies at King Khalid University, KSA, for funding this work through the Small Research Group under grant number (RGP.1/64/46).

## Ethics Statement

The study was approved by the Department of Mechatronics and Biomedical Engineering, Air University, Islamabad, Pakistan, with ethical approval number AU/EA/2025/606/18/4.

## Conflicts of Interest

The authors declare no conflicts of interest.

## Supporting information


**Figure S1:** The figure displays the performance stability of six machine learning classifiers across 10 folds. Subplots represent: (A) KNN, (B) Decision Tree, (C) Random Forest, (D) XGBoost, (E) LightGBM, and (F) CatBoost. The *y*‐axis represents Accuracy (0.0–1.0), and the *x*‐axis represents the Fold number. These results complement the primary findings in Figure 9 by illustrating the variance and consistency of all tested models, not solely the best‐performing CatBoost classifier.

## Data Availability

The data that support the findings of this study are available on request from the corresponding author. The data are not publicly available due to privacy or ethical restrictions.
