# Supplementary material for: AI‐Driven Hemodynamic Detection of Self‐Induced Daydreaming With EMG‐Based Physiological Triggers During Pre‐ and Post‐Prandial States Using fNIRS and EGG
Source: CNS Neurosci Ther. 2026 May 1;32(5):e70899. doi: 10.1002/cns.70899 (PMC13135031; doi:10.1002/cns.70899)
Supplement: Supplementary file 1 — Figure S1: The figure displays the performance stability of six machine learning classifiers across 10 folds. Subplots represent: (A) KNN, (B) Decision Tree, (C) Random Forest, (D) XGBoost, (E) LightGBM, and (F) CatBoost. The y‐axis represents Accuracy (0.0–1.0), and the x‐axis represents the Fold number. These results complement the primary findings in Figure 9 by illustrating the variance and consistency of all tested models, not solely the best‐performing CatBoost classifier. [file CNS-32-e70899-s001.docx]

**Supplementary**

Stratified 10-Fold Cross-Validation Accuracy Curves for f-NIRS-based Daydream Classification.

| a)  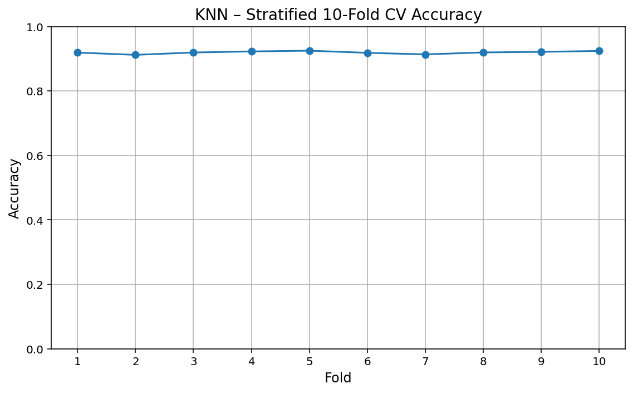 | b)  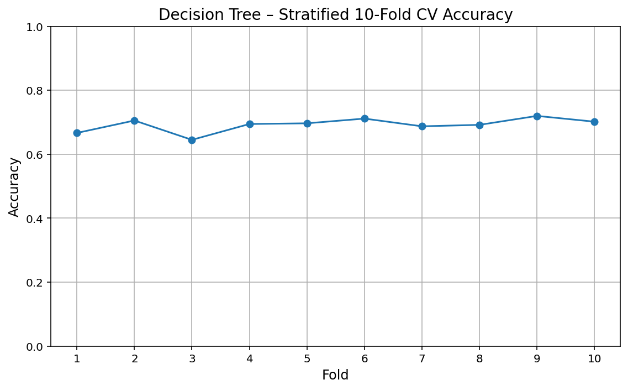 |
| --- | --- |
| c)  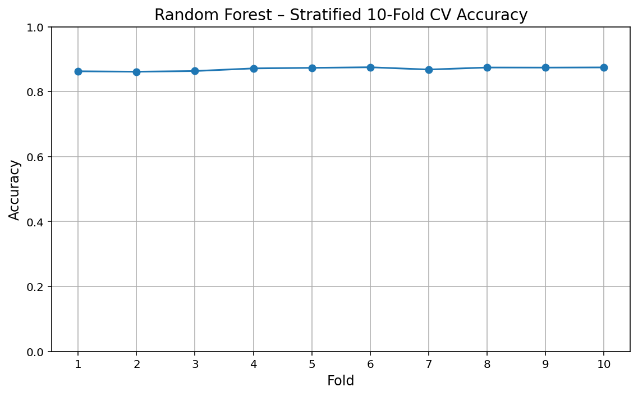 | d)  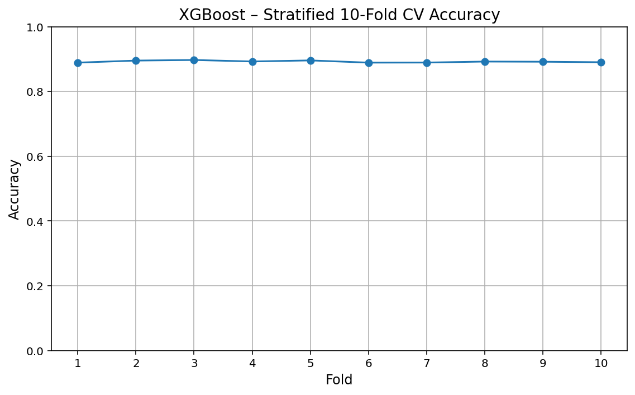 |
| e)  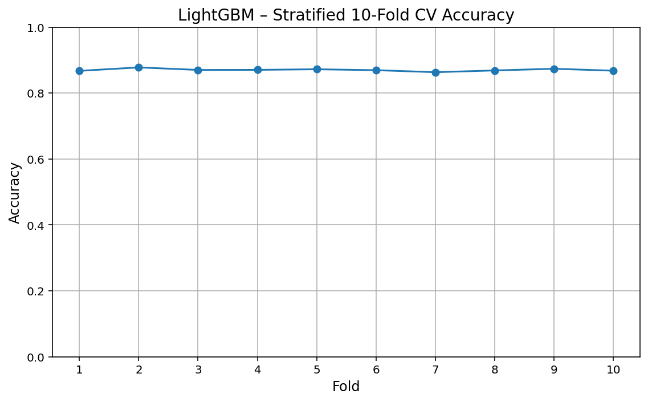 | f)  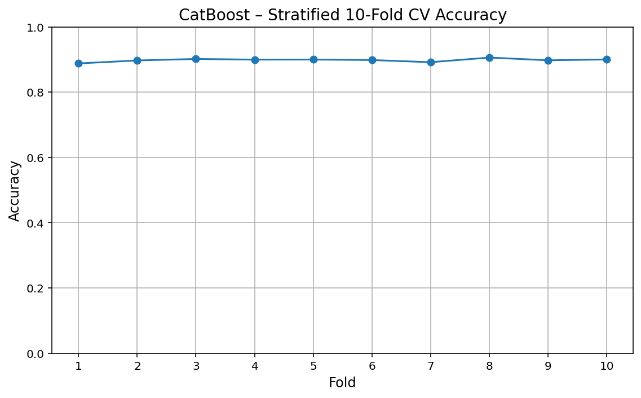 |

Figure S1 The figure displays the performance stability of six machine learning classifiers across 10 folds. Subplots represent: (A) KNN, (B) Decision Tree, (C) Random Forest, (D) XGBoost, (E) LightGBM, and (F) CatBoost. The Y-axis represents Accuracy (0.0 to 1.0), and the X-axis represents the Fold number. These results complement the primary findings in Figure 9 by illustrating the variance and consistency of all tested models, not solely the best-performing CatBoost classifier.
